# A generative model of hyperelastic strain energy density functions for multiple tissue brain deformation

**DOI:** 10.1007/s11548-020-02284-y

**Published:** 2020-11-09

**Authors:** Alejandro Granados, Fernando Perez-Garcia, Martin Schweiger, Vejay Vakharia, Sjoerd B. Vos, Anna Miserocchi, Andrew W. McEvoy, John S. Duncan, Rachel Sparks, Sébastien Ourselin

**Affiliations:** 1 School of Biomedical Engineering and Imaging Sciences, King’s College London, London, UK; 2 National Hospital for Neurology and Neurosurgery, London, UK; 3 Wellcome/EPSRC Centre for Interventional and Surgical Sciences, UCL, London, UK

**Keywords:** Biomechanics, Brain deformation, Gaussian Processes

## Abstract

**Purpose:**

Estimation of brain deformation is crucial during neurosurgery. Whilst mechanical characterisation captures stress–strain relationships of tissue, biomechanical models are limited by experimental conditions. This results in variability reported in the literature. The aim of this work was to demonstrate a generative model of strain energy density functions can estimate the elastic properties of tissue using observed brain deformation.

**Methods:**

For the generative model a Gaussian Process regression learns elastic potentials from 73 manuscripts. We evaluate the use of neo-Hookean, Mooney–Rivlin and 1-term Ogden meta-models to guarantee stability. Single and multiple tissue experiments validate the ability of our generative model to estimate tissue properties on a synthetic brain model and in eight temporal lobe resection cases where deformation is observed between pre- and post-operative images.

**Results:**

Estimated parameters on a synthetic model are close to the known reference with a root-mean-square error (RMSE) of 0.1 mm and 0.2 mm between surface nodes for single and multiple tissue experiments. In clinical cases, we were able to recover brain deformation from pre- to post-operative images reducing RMSE of differences from 1.37 to 1.08 mm on the ventricle surface and from 5.89 to 4.84 mm on the resection cavity surface.

**Conclusion:**

Our generative model can capture uncertainties related to mechanical characterisation of tissue. When fitting samples from elastography and linear studies, all meta-models performed similarly. The Ogden meta-model performed the best on hyperelastic studies. We were able to predict elastic parameters in a reference model on a synthetic phantom. However, deformation observed in clinical cases is only partly explained using our generative model.

**Electronic supplementary material:**

The online version of this article (10.1007/s11548-020-02284-y) contains supplementary material, which is available to authorized users.

## Introduction

Brain deformation during image-guided interventions could lead to errors in delivering treatment which may reduce efficacy or cause adverse events. There are many methods to predict tissue deformation by modelling mechanical behaviour. Whilst these models have been used for simulation and computer-aided interventions [15], tissue behaviour is often modelled using average values from the literature. To characterise tissue mechanical behaviour, observations of strain (displacement) during controlled application of stresses (forces) are recorded in vivo, i.e. magnetic resonance elastography (MRE) [11], or ex vivo, i.e. mechanical loading of resected tissue [6, 20]. Constitutive models capture stress–strain observations from these studies by assuming a known parametric model and optimising parameters to best describe the observed behaviour. However, there is large variability in reported data caused by many factors including tissue complexity (heterogeneity), limited tissue samples, inter-patient differences, and varying protocols [15]. Moreover, reported values in the literature may lead to numerical instability in models. Stability conditions require convex strain energy density functions (Supplemental Material (SM) Table 2) [7] with monotonic increasing of strain energy density with increasing strain (Drucker stability criterion; SM Fig. 2) [16, 28].

The work presented here is based on the generative model proposed in [10] where Gaussian process (GP) regression was used to learn distributions of strain energy density functions $\varPsi$ for brain tissue that account for aleatory and epistemic uncertainties of parameters estimated from mechanical characterisation studies. These distributions are sampled and used to determine the parameters of a neo-Hookean meta-model, constrained to guarantee valid $\varPsi$ on a wider range of strains than those performed during experiments. The aim of this work is to (1) extend our approach with two additional meta-models (Mooney–Rivlin and 1-term Ogden) and allow for multiple tissue parameter estimation (grey and white matter), and (2) apply learnt distributions of $\varPsi$ to the simulation of tissue deformation to predict patient-specific parameters to explain the observed strain–stress response. We expand our validation using a synthetic model to evaluate whether the generative model can recover the parameters of a known reference model, and on eight temporal lobe resection cases to demonstrate, we can simulate deformation observed on post-operative imaging.

## Related works

Constitutive models are expressed mathematically in closed form and consist of a variable number of parameters (neo-Hookean, Mooney–Rivlin, Ogden), are exponential (Demiray), or account for rapidly strain-stiffening behaviour (Gent, Fung). These models are phenomenological and parameters are typically adjusted to fit observations from mechanical loading experiments (e.g. compression, tension, shear, combined). Nonlinear least-squares optimisation is typically used to minimise the squared differences between experimentally determined and model predicted first Piola–Kirchhoff stresses [6]. Goodness of fit is evaluated using the coefficient of determination $R^2$.

However, high variability is common in experimental data resulting in uncertainties that constitutive models should take into account. Bayesian techniques have been proposed to model sources of uncertainties including aleatory (measurement noise) and epistemic uncertainty (inability to ascertain the validity of the chosen model and related parameters) [14, 16]. Reference [25] reviewed Bayesian inference techniques for material elastic properties and presented a framework for stochastic identification of elastic parameters for a 1D isotropic string. Reference [14] proposed a Bayesian strategy to directly infer stresses/pressure in the context of elastography whereby they acquire maximum *a posteriori* estimates of the discrepancies in model parameters using an expectation-maximisation algorithm whilst fully sampling remaining parameters from the posterior. Reference [16] proposed a Bayesian calibration framework to account for variability in the mechanical characterisation of soft tissue for aleatory and epistemic uncertainties. Reference [18] used an evolutionary algorithm to estimate elastic parameters of hyperelastic models with a geometric similarity function used as an optimisation criteria. Reference [1] proposed an inverse model for elastic parameter estimation of a bioprosthetic valve using its deformed state to fit exponential constitutive models. They observed that the objective function contained a long, and narrow valley in the parameter space. Parameters along this valley generate similar stress–strain responses. Reference [19] estimated elastic properties of porcine eyes using a Reduced-order Unscented Kalman filter [22].

## Methods

### Mechanical characterisation of human brain tissue

Biological tissue is commonly characterised by nonlinear hyperelastic models [20]. In contrast to linear models, the stress–strain relationship of a hyperelastic model is described through a phenomenological approach using a strain energy density function $\varPsi$ that is written in terms of the deformation gradient $\pmb {F}$ [17]. $\varPsi$ can be defined with principal invariants $I_c, II_c, III_c$ (SM Eq. 1) or principal stretches $\lambda _i$ (rotation invariant SVD of $\pmb {F}=\pmb {U}\hat{\pmb {F}}\pmb {V}^\mathrm{T}$, where $\hat{\pmb {F}}=\mathrm{diag}(\lambda _1, \lambda _2, \lambda _3)$) [27]. In this study three models are considered (Eq. 1): Neo-Hookean ($\varPsi _{\mathrm{NH}}$), Mooney–Rivlin ($\varPsi _{\mathrm{MR}}$) and 1-term Ogden ($\varPsi _{\mathrm{O}_1}$). Similar to [10], we gathered 73 models from MRE, linear, and hyperelastic studies found in the literature (SM Fig. 1) that characterised healthy brain tissue (without distinguishing type), grey matter, white matter, and abnormal tissue assessed under varying amounts of strain applied during compression/tension tests. MRE and linear models were reformulated as hyperelastic functions as in [10].

1 $$\begin{aligned} \varPsi _{\mathrm{NH}}(\lambda _1, \lambda _2, \lambda _3)= & {} \frac{\mu }{2}(\lambda _1^2 + \lambda _2^2 + \lambda _3^2 - 3)\nonumber \\ \varPsi _{\mathrm{MR}}(\lambda _1, \lambda _2, \lambda _3)= & {} C_1(\lambda _1^2 + \lambda _2^2 + \lambda _3^2 - 3) + C_2(\lambda _1^2 \lambda _2^2 \nonumber \\&+ \lambda _2^2 \lambda _3^2 + \lambda _3^2 \lambda _1^2 - 3)\nonumber \\ \varPsi _{\mathrm{O}_1}(\lambda _1, \lambda _2, \lambda _3)= & {} \sum _{p=1}^{N=1}\frac{\mu _p}{\alpha _p}(\lambda _1^{\alpha _p} + \lambda _2^{\alpha _p} + \lambda _3^{\alpha _p} - 3) \end{aligned}$$

2 $$\begin{aligned}&ln~\varPsi _{{\mathcal {G}}{\mathcal {P}}_t}(\pmb {\lambda }_t) \sim {\mathcal {G}}{\mathcal {P}}(0, k(\pmb {\lambda }_t,\pmb {\lambda }_t'))\nonumber \\ f_{{\mathcal {G}}{\mathcal {P}}_t}(\pmb {\lambda }_t)= & {} ln~\varPsi _{{\mathcal {G}}{\mathcal {P}}_t}(\pmb {\lambda }_t) + \pmb {\epsilon }_t \end{aligned}$$

3 $$\begin{aligned}&\begin{bmatrix} f_{{\mathcal {G}}{\mathcal {P}}_1}(\pmb {\lambda }_t) \\ \vdots \\ f_{{\mathcal {G}}{\mathcal {P}}_T}(\pmb {\lambda }_T) \end{bmatrix}\nonumber \\&\sim \mathcal {N}\left( \begin{bmatrix} \pmb {0} \\ \vdots \\ \pmb {0} \end{bmatrix}, \pmb {B}\otimes \pmb {K} + \pmb {\sigma }_t^2 \pmb {I} \right) \end{aligned}$$

### Generative model

We use GP regression to learn distributions over $\varPsi$ from models obtained from the literature. GP regression is a non-parametric Bayesian approach to regress an output $\pmb {y}$ of a function *f* given the input $\pmb {x}$. That is, $\pmb {y}=f(\pmb {x})+\epsilon$, where we assume $f(\pmb {x})$ is a random variable with a particular distribution and $\epsilon$ is observation randomness [26]. $f(\pmb {x})$ is defined by a mean and a covariance (*kernel*) function. We learn $f(\pmb {x})$ in log space ($ln~\varPsi$) to aid optimisation, as elastic potentials vary significantly between models. The data from the literature (($\pmb {\lambda }_{i,n}$, $\pmb {\varPsi }_{i,n}$) $\mid$ $n \in N=73$, $i \in I=100$ interpolation points) are *unbalanced* and *heterotopic*. To consolidate three types of studies (MRE, linear and hyperelastic) across four regions of tissue (grey matter, white matter, healthy, and abnormal), we define $T=12$ tasks ($f_{{\mathcal {G}}{\mathcal {P}}_t}(\pmb {\lambda }_t)$ $\mid$ $t \in T$). Each task is a multiple output vector-valued function that takes as inputs stretches $\lambda _t$ (SM Fig. 1) and outputs $ln~\varPsi$. Observation randomness is modelled as Gaussian noise $\pmb {\epsilon }_t \sim \mathcal {N}(\pmb {0},\pmb {\sigma }_t^2)$ (Eq. 2). We assume correlation across regions and studies, and learn all models jointly. This is important for studies applying small strains that may benefit from learnt $\varPsi$ at wider strains. The multi-task GP is defined as an *Intrinsic Coregionalisation Model (ICM)* [2], where covariance across tasks is mapped with a coregionalisation matrix $\pmb {B}=\pmb {W}\pmb {W}^\mathrm{T} + \pmb {\kappa }_t\pmb {I}$, where $\pmb {W}$ comprises task coefficients and $\kappa$ reflects variance across tasks (Eq. 3). To estimate covariance within tasks, we use a *Matérn 3/2* covariance kernel $k(\pmb {\lambda }_t,\pmb {\lambda }_t') = k_{M3/2}$. Kernels including squared exponential, linear, bias and simple combinations of these were tested empirically and $k_{M3/2}$ was found to best describe the data. The GP regression optimisation task has 38 parameters ($k_{M3/2}$ length scale and variance, 12-valued vectors $\pmb {W}$ (rank 1), $\pmb {\kappa }_t$ and $\pmb {\sigma }_t^2$).

### Hyperelastic meta-model

To guarantee GP distributions are stable over a wider range of strains we fit a meta-model function, either neo-Hookean ($\varPsi _{\mathrm{NH}}$), Mooney–Rivlin ($\varPsi _{\mathrm{MR}}$), or 1-term Ogden, ($\varPsi _{\mathrm{O}_1}$) (Eq. 1) to obtain a final stress–strain function. For a uniaxial tension/compression mechanical test, Eq. 1 can be reduced (SM Eq. 2). We use least squares optimisation of coefficient residuals in the form of $f_i(\lambda ) = ln (\varPsi _i + 0.001)$ using the Jacobian (SM Eq. 3) subject to stability conditions (SM Table 2) and bounds that we define after searching the parameter space for valid coefficients (SM Fig. 3).

**Fig. 1:**
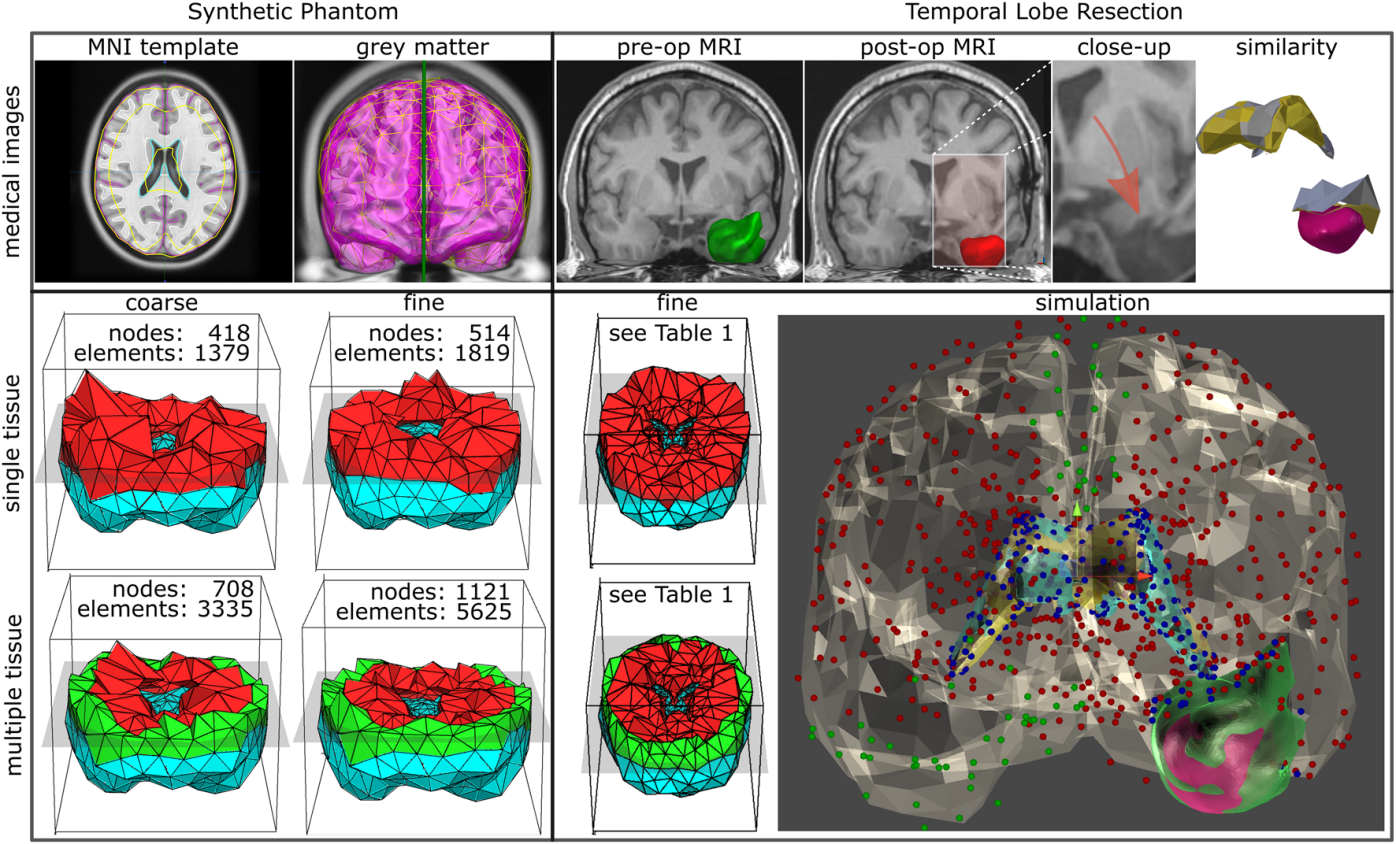
Experiments on a synthetic brain (left) and temporal lobe resection cases (right). RMSE is computed for all nodes of synthetic brain. Resection cases use nodes of the ventricles and resection margin. Simulation of resection cases includes brain (translucent), ventricles (deformed-yellow, reference-blue) and resection volume. Nodes set as fixed (green), under the influence of gravity (red and blue), and for similarity metric (blue) are highlighted

### Hyperelastic finite element method (FEM) simulation

We use a compressible neo-Hookean material in the form $\varPsi = \frac{\mu }{2}(\lambda _1^2 + \lambda _2^2 + \lambda _3^2 - 3) - \mu ln J + \frac{\lambda }{2}(ln J)^2$ (with *Lamé* coefficients $\mu$, $\lambda$, and principal stretches $\lambda _{i=1,2,3}$). We incorporate a volume preserving force, a compression resistance term added to $\varPsi$ when $J = \lambda _1 \lambda _2 \lambda _3 < 1$ [28]. We use implicit backward Euler integration to allow for large time steps [3]. The motion of the deformable solid, discretised into a tetrahedral mesh consisting of *n* nodes, is described by the Euler–Lagrange equation $\pmb {M} \pmb {\ddot{u}} + \pmb {D}(\pmb {u},\pmb {\dot{u}}) + \pmb {R}(\pmb {u}) = \pmb {f}$ where $\pmb {u} \in {\mathbb {R}}^{3n}$ is the unknown displacement vector, $\pmb {M} \in {\mathbb {R}}^{3n \times 3n}$ is the mass matrix, $\pmb {D}(\pmb {u},\pmb {\dot{u}}) \in {\mathbb {R}}^{3n \times 3n}$ are damping (Rayleigh) forces, $\pmb {R}(\pmb {u}) \in {\mathbb {R}}^{3n}$ are internal forces, and $\pmb {f} \in {\mathbb {R}}^{3n}$ are external forces (SM Table 4). A stiffness matrix $\pmb {K}(\pmb {u}) \in {\mathbb {R}}^{3n \times 3n}$ is computed as the Jacobian of $\pmb {R}(\pmb {u})$ and its nonlinear mapping is computed as the $1^{st}$ Piola–Kirchhoff stress tensor of the rotation invariant SVD (“Mechanical characterisation of human brain tissue” section) using the gradient and Hessian of $\varPsi$ for each element [27]. We use a modified conjugate gradient solver with a Jacobi preconditioner to solve for $\varDelta \pmb {\dot{u}}$ and compute $\pmb {u}=h(\pmb {\dot{u}_0} + \varDelta \pmb {\dot{u}})$ as in [3], where *h* indicates the time step.

### Evaluation

We demonstrate the validity and usability of our generative model on single tissue experiments, considering all elastic models except abnormal tissue, and multiple tissue experiments, considering only hyperelastic models for grey and white matter. We evaluate our experiments on root-mean-square error (RMSE) computed as $\mathrm{RMSE}(\mathbf{x }^{\mathrm{state}}, \mathbf{x }^{\mathrm{ref}}) = \sqrt{\frac{\sum _{i=1}^{S}(\mathbf{x }_i^{\mathrm{state}}-\mathbf{x }_i^{\mathrm{ref}})^2}{S}}$, where *S* is the number of similarity nodes and $x_i^{\mathrm{state}}$ the position of a node *i* for a given *state*.

*Validation* We evaluate our generative model using a synthetic model comparing a model with known reference parameters (*reference state*) and the same model with elastic parameters obtained from our generative model (*deformed state*). Both models undergo deformation following “Hyperelastic finite element method (FEM) simulation” section. The synthetic model with no deformation is defined as the *rest state*.

Our synthetic model is constructed using the 152 MNI template T1-weighted (T1-w) MRI [8] (Fig. 1 left). Deformations are performed in a controlled simulation environment with known boundary conditions and external forces. The base of the brain and the interaction of the brain with the falx cerebri are defined as Dirichlet boundary conditions whereby nodes within these regions are fixed (displacement is set to zero) [23]. An external force of 10 N is applied for compression to a subset of nodes located superiorly (SM Fig. 6).

To obtain a reference state, we assign for single tissue experiments the shear modulus ($\mu$) to 333.28 Pa [21], and for multiple tissue experiments a $\mu$ for grey matter to 1370 Pa (basal ganglia) and for white matter to 990 Pa (corpus callosum) [6]. To evaluate our generative model, we assume the reference tissue properties are unknown. We then sample possible $\varPsi$ from the ${\mathcal {G}}{\mathcal {P}}$ distribution ($\pm 2$ standard deviations (SD)), fit them to a neo-Hookean meta-model, and deform the synthetic model. We then compare reference and deformed states computing $\mathrm{RMSE}(\mathbf{x }^{\mathrm{def}}, \mathbf{x }^{\mathrm{ref}})$ between models to identify the meta-model that best fits the observed deformation of the reference state.

*Usability* We assess the ability of our generative model to estimate elastic properties in a real-world scenario where brain tissue, as observed on pre-operative imaging (*rest state*), has undergone deformation, as observed on post-operative imaging, after temporal lobe resection (*reference state*). We compare the reference state with a model obtained by deforming the rest state (following “Hyperelastic finite element method (FEM) simulation” section) using our generative model (*deformed state*).

Rest and reference states of eight patients are constructed using pre- and post-operative T1-w MRI rigidly registered to MNI space [8] (SM Fig. 7). The resection cavity is automatedly segmented on post-operative images [24] and the resected tissue is manually delineated in pre-operative images (Fig. 1 right). We then subtract the resected tissue identified manually from the rest state. As in the synthetic experiment, the base of the brain and the interaction of the brain with the falx cerebri are defined as Dirichlet boundary conditions. Gravity is applied to all nodes as external forces.

To obtain a deformed state, we sample possible strain energy density functions from the ${\mathcal {G}}{\mathcal {P}}$ distribution ($\pm 2$ SD), fit them to a neo-Hookean meta-model, and deform the rest state. We then compare $\mathrm{RMSE}(\mathbf{x }^{\mathrm{rest}},\mathbf{x }^{\mathrm{ref}})$ of nodes located along resection margins and ventricles between reference and rest states, and $\mathrm{RMSE}(\mathbf{x }^{\mathrm{def}},\mathbf{x }^{\mathrm{ref}})$ between the reference and deformed states.

**Fig. 2:**
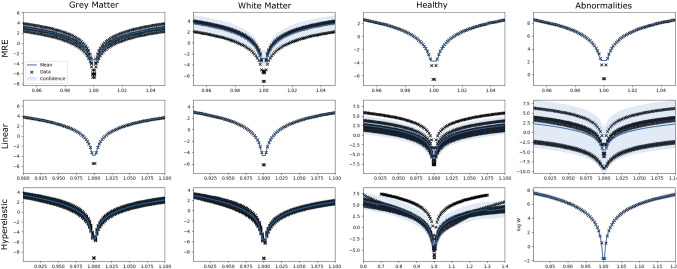
GP regression for 12 $\varPsi$ tasks (log space). Each figure shows stretches (*x*-axis) versus $\varPsi$ in log scale (*y*-axis). Black crosses indicate data from the literature, whereas blue solid lines and light shaded areas indicate mean and confidence interval, respectively

**Fig. 3:**
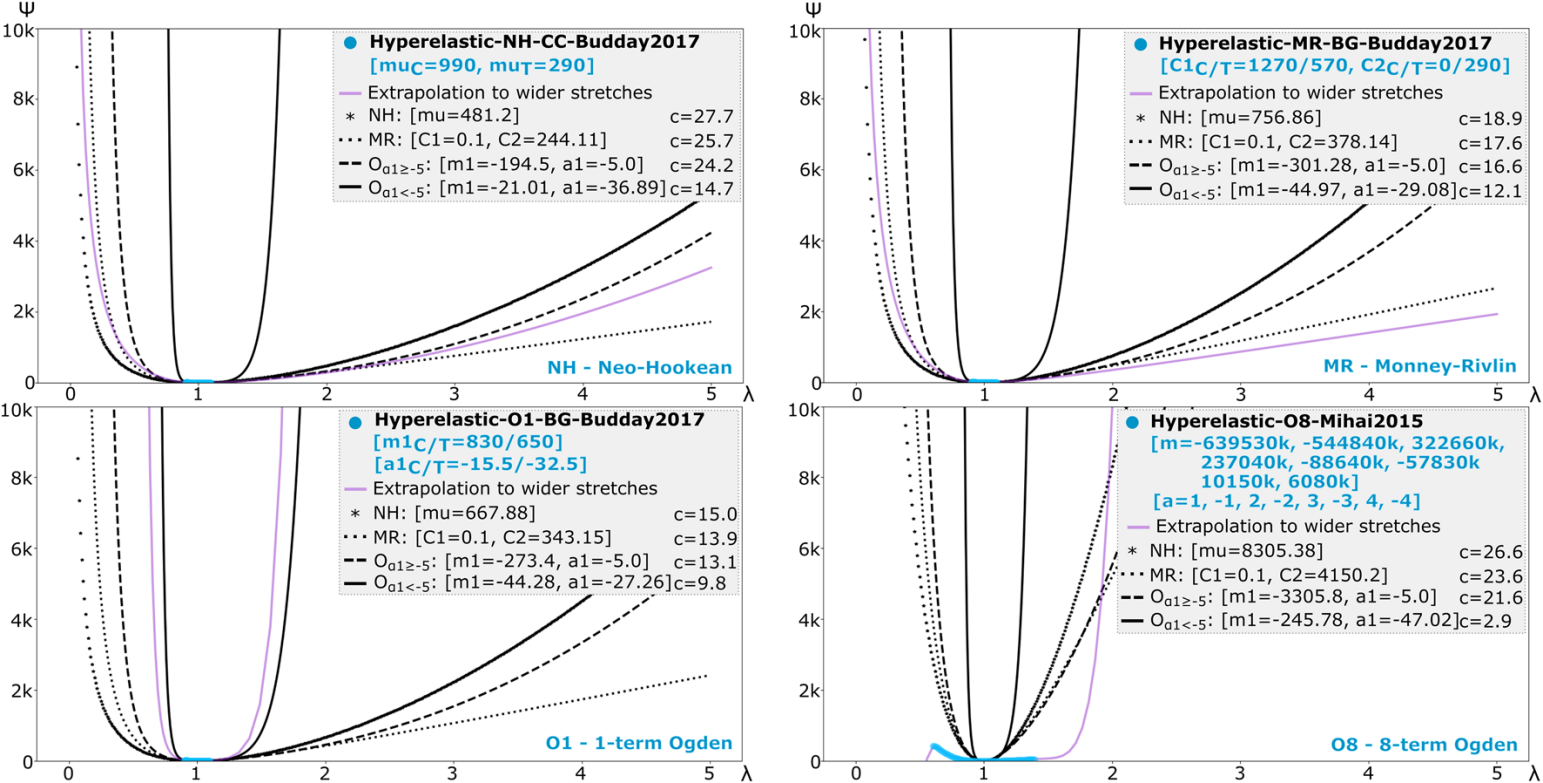
Evaluation of meta-models on their ability to represent constitutive models found in the literature. Each figure shows a model ($\varPsi$) from the literature (blue—within reported range of stretches; purple—over a wider window) fit by neo-Hookean (NH), Mooney–Rivlin (MR), and 1-term Ogden ($\mathrm{O}_{\alpha _1\ge -5.0}$ and $\mathrm{O}_{\alpha _1<-5.0}$) meta-models (black). Model parameters are shown within brackets for the literature model (blue) and those determined by the meta-models. Associated optimisation costs (*c*) are shown. Subscripts ‘C’ and ‘T’ indicate values reported for compression and tension, respectively

### Implementation

The generative model is implemented in GPy. Mechanical tests are evaluated using symbolic mathematics (SymPy). Medical images are loaded (NiBabel/SITK), registered (NiftiReg), and processed (TetGen) to generate triangular and four-noded tetrahedral meshes. Least square minimisation of the meta-model is performed with the Trust Region Reflective method (SciPy). For real-time simulation, we implemented a hyperelastic FEM partly based on VegaFEM [4] and Stomakhin et al. [27] as a native C++ plugin in Unity3D (https://unity.com/). We use multi-threading and GPU processing so the deformation of brain tissue is generated in real-time, excluding pre-processing time to segment the brain and related structures.

**Table 1:** Estimated elastic properties in temporal lobe resection cases

Patient				Deformation state									
		Rest state		Single tissue				Multiple tissue					
		Ventricles	Resection	Ventricles		Resection		Ventricles			Resection		
Case	Temporal lobe	RMSE	RMSE	mu ${\mathcal {G}}{\mathcal {P}}_t$	RMSE	mu ${\mathcal {G}}{\mathcal {P}}_t$	RMSE	$\mu _{gm}$ SD	$\mu _{wm}$ SD	RMSE	$\mu _{gm}$ SD	$\mu _{wm}$ SD	RMSE
0529	left	0.94	5.86	2746	**0.92**	90	**5.36**	1317	1195	**0**.**92**	525	309	**5.24**
				lin-gm		hyp-nh		0.5	2.0		− 2.0	− 2.0	
0614	left	1.58	4.69	519	**1.49**	142	**3.77**	2299	440	**1**.**51**	628	309	**3.70**
				hyp-wm		hyp-nh		2.0	− 1.0		− 1.5	− 2.0	
0660	left	1.41	5.83	440	**1.07**	257	**5.15**	525	309	**1**.**04**	525	309	**4.98**
				hyp-wm		lin-nh		− 2.0	− 2.0		− 2.0	− 2.0	
0685	left	1.25	5.57	754	**1.06**	142	**4.53**	1317	365	**1**.**05**	525	309	**4.63**
				hyp-gm		hyp-nh		0.5	− 1.5		− 2.0	− 2.0	
0535	right	1.61	6.42	628	**1.44**	79	**4.16**	754	440	**1**.**42**	525	309	**4.94**
				hyp-gm		lin-nh		− 1.0	− 1.0		− 2.0	− 2.0	
0555	right	1.33	7.43	465	**1.11**	79	**5.25**	912	309	**1**.**07**	628	309	**5.98**
				lin-nh		lin-nh		− 0.5	− 2.0		− 1.5	− 2.0	
0603	right	1.14	5.92	1317	**1.06**	79	**4.53**	2299	610	**1**.**08**	525	309	**4.75**
				hyp-gm		lin-nh		2.0	0.0		− 2.0	− 2.0	
0684	right	1.95	8.77	353	**1.62**	79	**5.69**	525	309	**1**.**61**	525	309	**6.91**
				hyp-nh		lin-nh		− 2.0	− 2.0		− 2.0	− 2.0	
Median		1.37	5.89	574	1.09	85	4.84	1115	403	1.08	525	309	4.96
MAD		0.22	0.42	157	0.19	6	0.47	475	94	0.1	0	0	0.31

$\mathrm{RMSE}(\mathbf{x }^{\mathrm{rest}},\mathbf{x }^{\mathrm{ref}})$, computed between the *rest* (no deformation has been performed) and *reference* states, is used as a benchmark

Our model captures post-operative changes in the brain when $\mathrm{RMSE}(\mathbf{x }^{\mathrm{def}},\mathbf{x }^{\mathrm{ref}})$ is lower than $\mathrm{RMSE}(\mathbf{x }^{\mathrm{rest}},\mathbf{x }^{\mathrm{ref}})$, indicated by bolded text. The best-performing model is underlined

For single tissue experiments we report estimated shear modulus $\mu$ and the corresponding distribution of $\varPsi$ from our generative model (${\mathcal {G}}{\mathcal {P}}_t$)

In multiple tissue experiments, we report shear modulus of grey ($\mu _{gm}$) and white ($\mu _{wm}$) matter, and the SD used to sample from the corresponding distributions

Median and median absolute deviation (MAD) of values over all 8 cases are shown in the last row

**Fig. 4:**
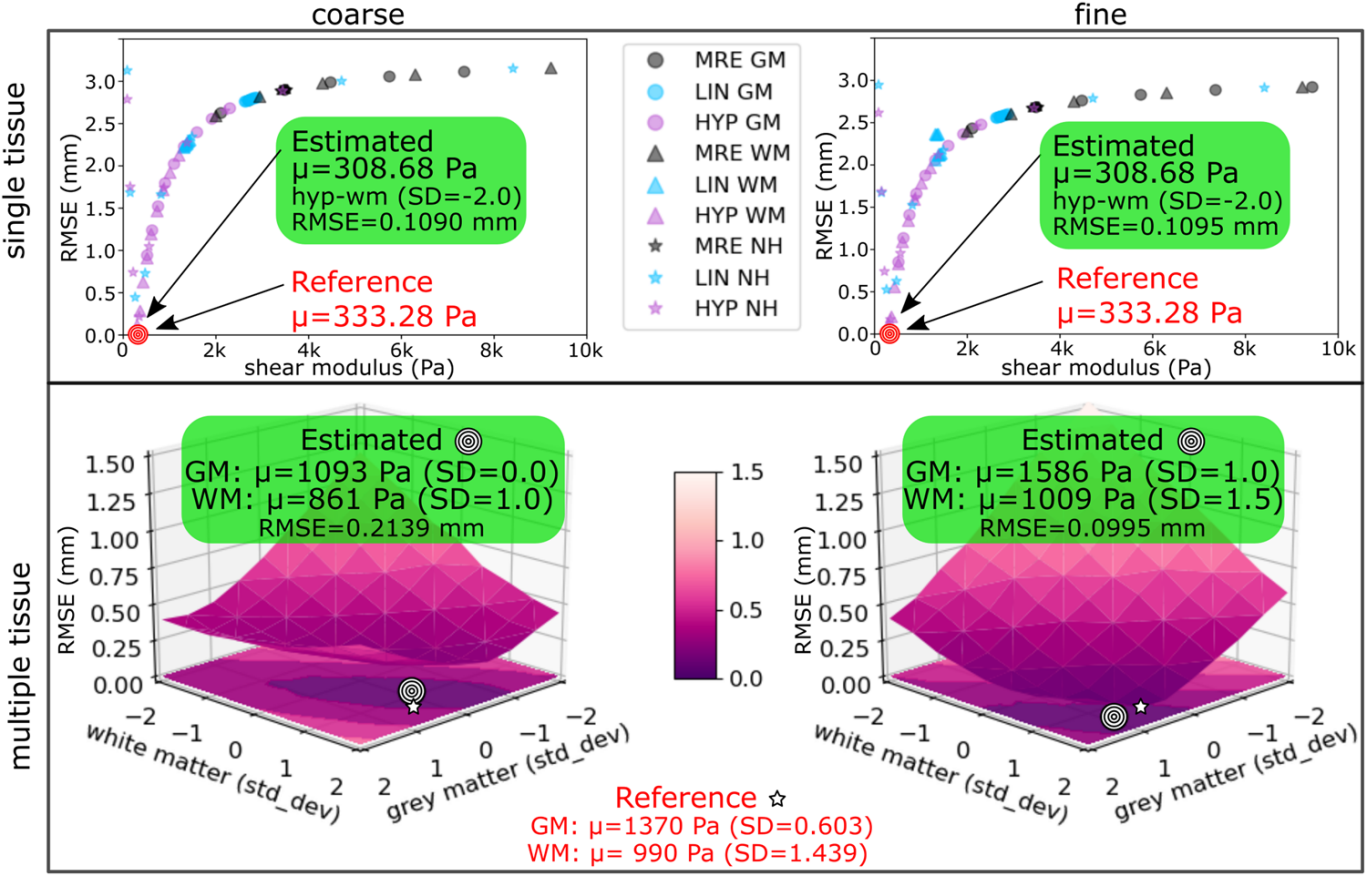
Validation on a synthetic phantom. RMSE is computed between a *reference state* (constructed using known elastic properties) and a *deformed state* (constructed using our generative model ($\pm 2$ SD)). Estimated elastic properties, corresponding to the sample with the lowest RMSE, are close to the known reference values

**Fig. 5:**
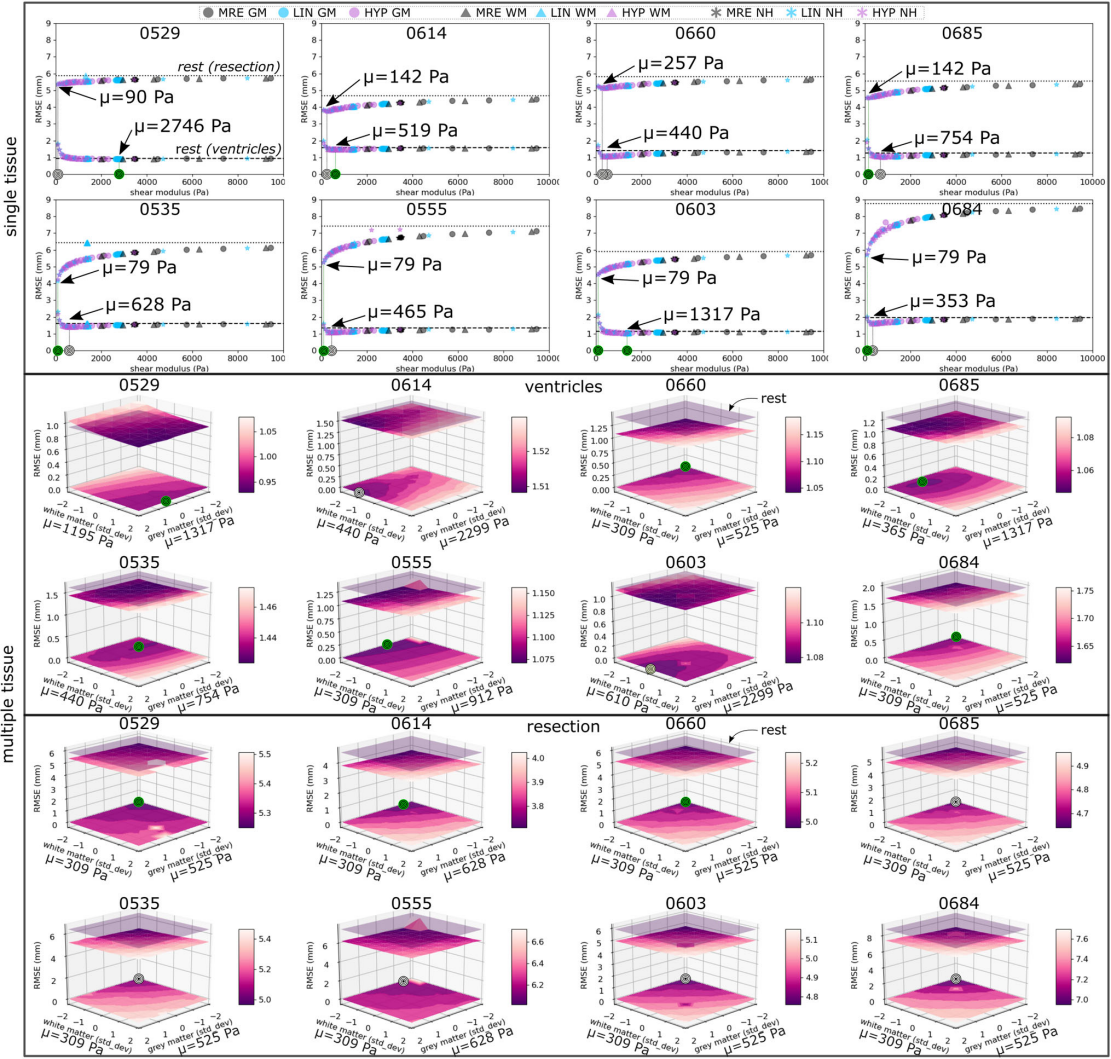
Validation on eight temporal lobe resection cases plotting RMSE of single and multiple tissue experiments. Best-performing cases are highlighted (green circle). *Single tissue* $\mathrm{RMSE}(\mathbf{x }^{\mathrm{rest}}, \mathbf{x }^{\mathrm{ref}})$ is shown as horizontal lines for ventricles (dashed) and resection margins (dotted). Each distribution was sampled 9 times ($\pm 2$ SD)). *Multiple tissue* $\mathrm{RMSE}(\mathbf{x }^{\mathrm{rest}}, \mathbf{x }^{\mathrm{ref}})$ is shown as a horizontal plane (translucent in purple). Distributions of grey and white matter were sampled 81 times ($\pm 2$ SD). $\mathrm{RMSE}(\mathbf{x }^{\mathrm{def}}, \mathbf{x }^{\mathrm{ref}})$ is plotted as a surface, projected onto the bottom plane. Note colour bars in each plot have different scales

## Results

### Generative model and hyperelastic meta-models

The ability of the GP model to learn distributions of $\varPsi$ in log scale is shown in Fig. 2. Studies of abnormal brain tissue are the most varying, likely due to the range of pathologies measured. We consider four meta-models to optimise their parameters when fitting models from the literature: neo-Hookean ($\varPsi _{\mathrm{NH}}$), Mooney–Rivlin ($\varPsi _{\mathrm{MR}}$), and 1-term Ogden meta-models with either a high $a_1$ ($\varPsi _{\mathrm{O}_{\alpha _1\ge -5.0}}$) or a low $a_1$ ($\varPsi _{\mathrm{O}_{\alpha _1<-5.0}}$) (see Eq. 1). All meta-models performed equally when sampling functions from MRE and linear distributions, regardless of tissue type. However, a Kruskal–Wallis nonparametric test, with Bonferroni correction, indicated statistical differences across all meta-models of hyperelastic distributions for grey matter ($H=14.59$; $p=0.001$), white matter ($H=20.98$; $p<0.001$), and healthy brain ($H=15.17$; $p=0.001$), with $\varPsi _{\mathrm{O}_{\alpha _1<-5.0}}$ performing the best followed by $\varPsi _{\mathrm{O}_{\alpha _1\ge -5.0}}$, $\varPsi _{\mathrm{MR}}$ and $\varPsi _{\mathrm{NH}}$.

To demonstrate the performance of meta-models in specific examples, we select four models from the literature that are more complex (i.e. different parameters for compression (*C*) and tension (*T*) or higher number of terms): neo-Hookean (NH), Mooney–Rivlin (MR), 1-term Ogden (O1), and 8-term (O8) Ogden models (Fig. 3). For the *NH* model, all meta-models, except $\varPsi _{\mathrm{O}_{\alpha _1<-5.0}}$, performed similarly. The $\varPsi _{\mathrm{MR}}$ meta-model better fit the MR model compared to other meta-models. The $\varPsi _{\mathrm{O}_{\alpha _1<-5.0}}$ meta-model only fit well the O1 model from the literature. None of the meta-models were able to fit O8.

### Evaluation

Elapsed time of key steps is computed when simulating the deformation of the brain phantom. Single tissue experiments execute in 6–8 Hz with performance decreasing to 1.5–3 Hz in multiple tissue experiments (SM Table 3).

*Validation on MNI phantom* In all experiments, estimated properties were close to the known reference parameter(s) (Fig. 4). However, we observe a valley where material properties of two tissue types have high covariance, similar to the parameter valley described in [1].

*Application in resection* Table 1 summarises the experimental results (Fig. 5). In single tissue experiments our generative model was unable to account for the deformation of ventricles with only minor improvements in RMSE in all cases. Multiple tissue experiments in general outperformed single tissue experiments for ventricles, where RMSE values were lower between deformed and reference states compared to the rest and reference states. For the resection margins, improvements in RMSE were observed in all cases, with lower shear modulus observed in the best performing cases. However, multiple tissue experiments outperformed single tissue experiments in only three cases.

## Discussion

Mechanical characterisation of soft tissue is unable to make predictions outside the calibrated range, a problem referred as model variance [16]. In this work, the use of a meta-model optimised on samples from distributions of $\varPsi$ guarantees stable predictions over a wider range of stretches for simulation. We evaluated a neo-Hookean meta-model for how our generative model can be validated and applied to clinical cases. We put special emphasis on achieving (near) real-time simulation. This work highlights the challenges towards a framework whereby *plug and play* elastic models could be chosen from a distribution of strain energy density functions to model a patient-specific case.

The simulation of soft tissue in clinical scenarios is a challenging problem and characterised by a discretisation of the domain of interest and the need to assume, with limited knowledge, boundary conditions and external forces. In this work we assumed fixed nodes to model the interaction of the brain with the falx cerebri. However, other works have suggested bilateral constraints may be more appropriate to model the interactions [23]. Additionally, brain tissue is mechanically characterised under scenarios that are different from those observed during/following surgical intervention which makes applying these models to surgical planning and simulation difficult. Without ground truth, we assessed the ability of our generative model to estimate parameters that could explain the observed deformation in temporal lobe resection cases. We selected a metric that considered nodes at the ventricles and along resection margins, as these regions are the most visible landmarks for evaluation.

There are two key limitations in our work. First, the use of low resolution meshes, treating ventricles as cavities, and assumptions of boundary conditions all may effect the model deformations. The quality of our generative model is only as good as that of the biomechanical model. However, there is no clear rule to select these for patient-specific cases. A better understanding of the forces exerted on tissue during/after surgery is required to more accurately simulate tissue deformation since motion does not necessarily correlate with gravity [13]. We use gravity as a heuristic based on [9] which suggested it is the primary cause of local sagging in tissue. We observe that ventricles moved sideways towards the hemisphere that had the resection (SM Fig. 7) and, on average, their volumes increased 23.1% between pre- and post-operative images. We hypothesise that the fall of the tissue into the resection cavity is due to the fact that the cerebrospinal fluid is sucked out during resection. Then, when the fluid fills the cavity, the tissue remains in position. Second, the models and values used to construct our GP distributions may not be able to explain the tissue deformation observed between pre- and post-operative images. Our results indicate that estimated tissue parameters may lie outside the GP distributions of hyperelastic grey/white matter. Despite this, improvements in RMSE in the resection margin were consistent across experiments.

## Conclusions and future work

We extended our generative model presented in [10] for this work by incorporating additional meta-models and multiple tissue sampling. We validated this extended model on its ability to estimate elastic properties given a known reference and explain the deformation observed in temporal lobe resections. Further work is necessary to use our generative model as a framework to estimate elastic parameters with optimised search strategies that are not uniform and to improve the simulation environment. For instance, although the compression resistance term used in the hyperelastic FEM is a heuristic to reduce volumetric locking, a common occurrence in tetrahedra elements, it does not guarantee volume preservation. Whilst this can be improved with incompressible models that alleviate volumetric locking [5, 12], in this work we focused on sampling values from Gaussian process distributions regressed from studies in the literature. More importantly, further investigation is necessary to improve our methodology on clinical cases and demonstrate its potential benefits for neurosurgical procedures intra-operatively that can be affected by brain shift, especially in cases where pathology is present [9].

## Electronic supplementary material

Below is the link to the electronic supplementary material.

Supplementary material 1 (pdf 13003 KB)
