# Supplementary material for: A generative model of hyperelastic strain energy density functions for multiple tissue brain deformation
Source: Int J Comput Assist Radiol Surg. 2020 Nov 9;16(1):141–50. doi: 10.1007/s11548-020-02284-y (PMC7822772; doi:10.1007/s11548-020-02284-y)
Supplement: Supplementary file 1 — Supplementary material 1 (pdf 13003 KB) [file 11548_2020_2284_MOESM1_ESM.pdf]

Noname manuscript No.
(will be inserted by the editor)

A Generative Model of Hyperelastic Strain Energy Density Functions for Multiple Tissue Brain Deformation

Supplemental Material

Alejandro Granados[1] · Fernando Perez-Garcia[3] · Martin Schweiger[1] · Vejay Vakharia[2] · Sjoerd B. Vos[2] · Anna Miserocchi[2] · Andrew W. McEvoy[2] · John S. Duncan[2] · Rachel Sparks[1] · Sébastien Ourselin[1]

Received: date / Accepted: date

Abstract *Purpose:* Estimation of brain deformation is crucial during neurosurgery. Whilst mechanical characterisation captures stress-strain relationships of tissue, biomechanical models are limited by experimental conditions. This results in variability reported in the literature. The aim of this work is to demonstrate a generative model of strain energy density functions can estimate the elastic properties of tissue using observed brain deformation. *Methods:* For the generative model a Gaussian Process regression learns elastic potentials from 73 manuscripts. We evaluate the use of Neo-Hookean, Mooney-Rivlin and 1-term Ogden meta-models to guarantee stability. Single and multiple tissue experiments validate the ability of our generative model to estimate tissue properties on a synthetic brain model and in eight temporal lobe resection cases where deformation is observed between pre- and post-operative images. *Results:* Estimated parameters on a synthetic model are close to the known reference with a root mean square error (RMSE) of 0.1 mm and 0.2 mm between surface nodes for single and multiple tissue experiments. In clinical cases, we were able to recover brain deformation from pre- to post-operative images reducing RMSE of differences from 1.37 mm to 1.08 mm on the ventricle surface, and from 5.89 mm to 4.84 mm on the resection cavity surface. *Conclusion:* Our generative model can capture uncertainties related to mechanical characterisation of tissue. When fitting samples from elastography and linear studies, all meta-models performed similarly. The Ogden meta-model performed the best on hyperelastic studies. We were able to predict elastic parameters in a reference model on a synthetic phantom. However, deformation observed in clinical cases is only partly explained using our generative model.

✉ E-mail: alejandro.granados@kcl.ac.uk
[1] School of Biomedical Engineering and Imaging Sciences, King's College London, UK
[2] National Hospital for Neurology and Neurosurgery, London, UK
[3] Wellcome/EPSRC Centre for Interventional and Surgical Sciences, UCL, UK

1 Elastic models of brain tissue from the literature

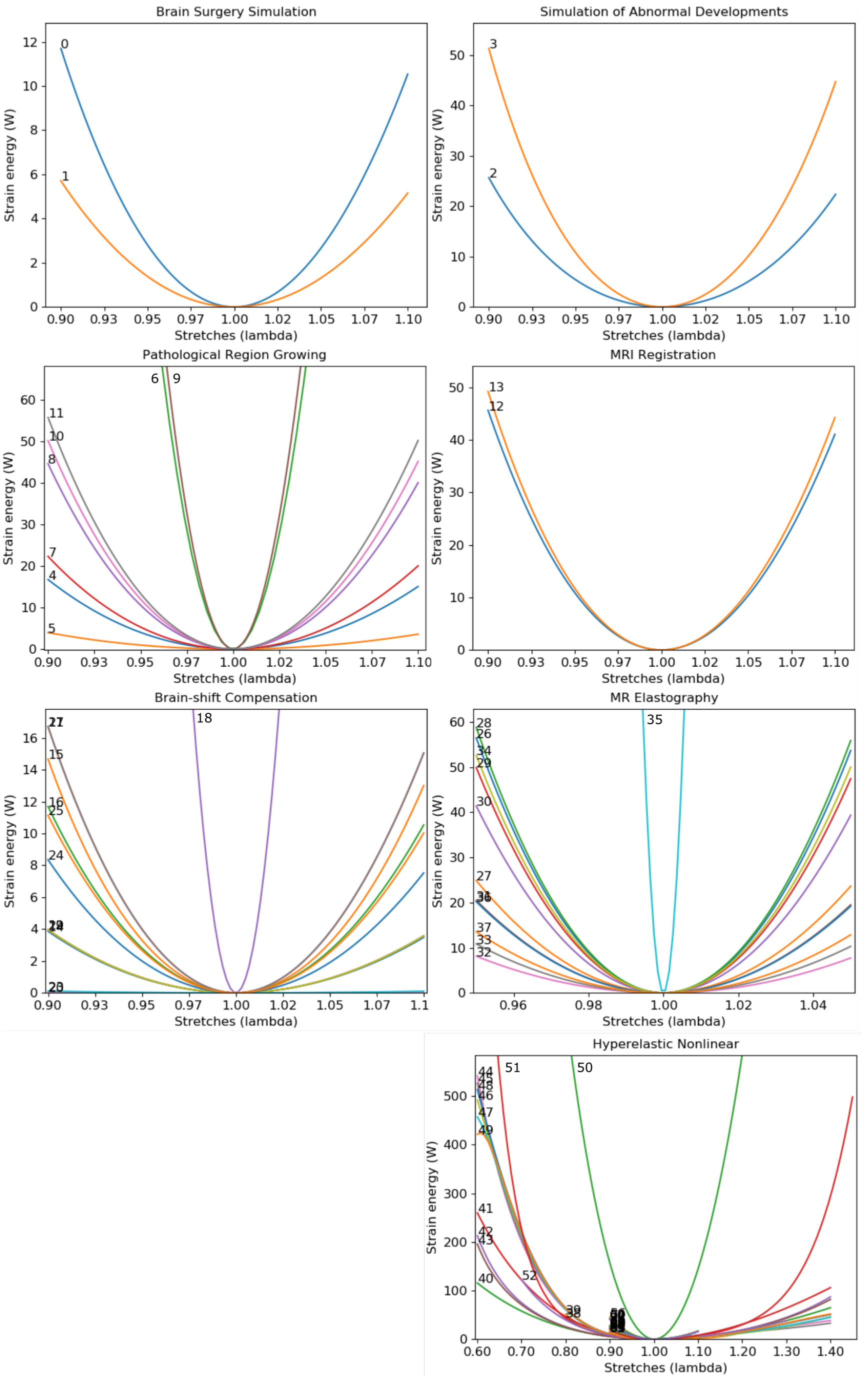

Fig. 1 Brain tissue models and parameters reported in the literature plotted as strain energy density functions. Horizontal axis refers to the amount of strain during rest ($\lambda_i = 1$), tension ($\lambda_i > 1$), and compression ($\lambda_i < 1$). Vertical axis refers to the elastic potential Ψ. Numbers correspond to column 'n' in Table 1.

Table 1 MRE, linear and hyperelastic studies in the literature reporting elastic parameters of grey matter, white matter, healthy brain and abnormalities. Rather than listing every study, especially numerous studies related to linear and MRE models, we refer to the survey presented by Morin et al (2017) [4], where the original references can be found. The exact parameters used from these studies can be located in the source code accompanying this manuscript. In the source code each study is referenced by the study name found in this table.

n	Study	Anatomy	Model
0	SurgSim-Dequidt2015 [4]	brain	linear
1	SurgSim-Sase2015 [4]	brain	linear
2	Abnormal-WM-castellanoSmith2003 [4]	abnormalities	linear
3	Abnormal-GM-castellanoSmith2003 [4]	abnormalities	linear
4	Pathos-Yousefi2013 [4]	brain	linear
5	Pathos-Prastawa2009 [4]	brain	linear
6	Pathos-TUMOUR-Kyriacou1999 [4]	abnormalities	hyperelastic
7	Pathos-WM-Takizawa1994 [4]	white matter	linear
8	Pathos-GM-Takizawa1994 [4]	grey matter	linear
9	Pathos-Tumour-Dumpuri2006 [4]	abnormalities	linear
10	BrainShift-tumour-Miller2013 [4]	abnormalities	linear
11	BrainShift-tumour-Morin2016 [4]	abnormalities	linear
12	Reg-min-Soza2004 [4]	brain	linear
13	Reg-max-Soza2004 [4]	brain	linear
14	BrainShift-Clatz2005 [4]	brain	linear
15	BrainShift-Wittek2009 [4]	brain	linear
16	BrainShift-Dumpuri2006 [4]	brain	linear
17	BrainShift-Vigneron2012 [4]	brain	linear
18	BrainShift-deLorenzo2012 [4]	brain	linear
19	BrainShift-Bucki2012 [4]	brain	linear
20	BrainShift-ventricles-Bucki2012 [4]	abnormalities	linear
21	BrainShift-Miller2013 [4]	brain	linear
22	BrainShift-Mohammadi2015 [4]	brain	linear
23	BrainShift-ventricles-Mohammadi2015 [4]	abnormalities	linear
24	BrainShift-Morin2016 [4]	brain	linear
25	BrainShift-Clatz2003 [4]	brain	linear
26	MRE-WM-Kruse1999 [4]	white matter	MRE
27	MRE-GM-Kruse1999 [4]	grey matter	MRE
28	MRE-WM-Uffmann2004 [4]	white matter	MRE
29	MRE-GM-Uffmann2004 [4]	grey matter	MRE
30	MRE-WM-McCracken2005 [4]	white matter	MRE
31	MRE-GM-McCracken2005 [4]	grey matter	MRE
32	MRE-WM-Green2006 [4]	white matter	MRE
33	MRE-GM-Green2006 [4]	grey matter	MRE
34	MRE-WM-Kruse2008 [4]	white matter	MRE
35	MRE-abnormal-WM-Kruse2008 [4]	abnormalities	MRE
36	MRE-GM-Kruse2008 [4]	grey matter	MRE
37	MRE-Hamhaber2007 [4]	brain	MRE
38	Hyperelastic-Ogden1-Mihai2017 [2]	brain	hyperelastic
39	Hyperelastic-MR3-Mihai2017 [2]	brain	hyperelastic
40	Hyperelastic-NH-Mihai2015 [3]	brain	hyperelastic
41	Hyperelastic-MR-Mihai2015 [3]	brain	hyperelastic
42	Hyperelastic-Fung-Mihai2015 [3]	brain	hyperelastic
43	Hyperelastic-Gent-Mihai2015 [3]	brain	hyperelastic
44	Hyperelastic-Ogden3-Mihai2015 [3]	brain	hyperelastic
45	Hyperelastic-Ogden4-Mihai2015 [3]	brain	hyperelastic
46	Hyperelastic-Ogden5-Mihai2015 [3]	brain	hyperelastic
47	Hyperelastic-Ogden6-Mihai2015 [3]	brain	hyperelastic
48	Hyperelastic-Ogden7-Mihai2015 [3]	brain	hyperelastic
49	Hyperelastic-Ogden8-Mihai2015 [3]	brain	hyperelastic
50	Hyperelastic-MR3-Laksari2012 [4]	brain	hyperelastic
51	Hyperelastic-MR2-Schiavone2009 [4]	brain	hyperelastic
52	Hyperelastic-OgdenMod1-Miller2002 [4]	brain	hyperelastic
53	Hyperelastic-NH-CC-Budday2017 [1]	white matter	hyperelastic
54	Hyperelastic-NH-CR-Budday2017 [1]	white matter	hyperelastic
55	Hyperelastic-NH-BG-Budday2017 [1]	grey matter	hyperelastic
56	Hyperelastic-NH-C-Budday2017 [1]	grey matter	hyperelastic
57	Hyperelastic-MR-CC-Budday2017 [1]	white matter	hyperelastic
58	Hyperelastic-MR-CR-Budday2017 [1]	white matter	hyperelastic
59	Hyperelastic-MR-BG-Budday2017 [1]	grey matter	hyperelastic
60	Hyperelastic-MR-C-Budday2017 [1]	grey matter	hyperelastic
61	Hyperelastic-DMR-CC-Budday2017 [1]	white matter	hyperelastic
62	Hyperelastic-DMR-CR-Budday2017 [1]	white matter	hyperelastic
63	Hyperelastic-DMR-BG-Budday2017 [1]	grey matter	hyperelastic
64	Hyperelastic-DMR-C-Budday2017 [1]	grey matter	hyperelastic
65	Hyperelastic-Gent-CC-Budday2017 [1]	white matter	hyperelastic
66	Hyperelastic-Gent-CR-Budday2017 [1]	white matter	hyperelastic
67	Hyperelastic-Gent-BG-Budday2017 [1]	grey matter	hyperelastic
68	Hyperelastic-Gent-C-Budday2017 [1]	grey matter	hyperelastic
69	Hyperelastic-Ogden1-CC-Budday2017 [1]	white matter	hyperelastic
70	Hyperelastic-Ogden1-CR-Budday2017 [1]	white matter	hyperelastic
71	Hyperelastic-Ogden1-BG-Budday2017 [1]	grey matter	hyperelastic
72	Hyperelastic-Ogden1-C-Budday2017 [1]	grey matter	hyperelastic

2 Strain energy density functions

2.1 Strain energy density functions in terms of invariants by means of the right Cauchy-Green deformation tensor $\boldsymbol{C} = \boldsymbol{F}^T\boldsymbol{F}$

$$
\begin{aligned}
I_c = trace(C) = \lambda_1^2 + \lambda_2^2 + \lambda_3^2 \\
II_c = C : C = \lambda_1^2\lambda_2^2 + \lambda_2^2\lambda_3^2 + \lambda_3^2\lambda_1^2 \\
III_c = detC = \lambda_1^2\lambda_2^2\lambda_3^2
\end{aligned}
\tag{1}
$$

2.2 Meta-models defined under uniaxial mechanical loading, where $\lambda_2 = \lambda_3 = \lambda_1^{-\frac{1}{2}}$ to ensure volume conservation ($J = \lambda_1\lambda_2\lambda_3 = 1$)

$$
\begin{aligned}
\Psi_{NH}(\lambda_1, \lambda_1^{-\frac{1}{2}}, \lambda_1^{-\frac{1}{2}}) = \frac{\mu}{2}(\lambda_1^2 + 2\lambda_1^{-1} - 3) \\
\Psi_{MR}(\lambda_1, \lambda_1^{-\frac{1}{2}}, \lambda_1^{-\frac{1}{2}}) = C_1(\lambda_1^2 + 2\lambda_1^{-1} - 3) + C_2(2\lambda_1 + \lambda_1^{-2} - 3) \\
\Psi_{O_1}(\lambda_1, \lambda_1^{-\frac{1}{2}}, \lambda_1^{-\frac{1}{2}}) = \sum_{p=1}^{N} \frac{\mu_p}{\alpha_p}(\lambda_1^{\alpha_p} + 2\lambda_1^{-\frac{\alpha_p}{2}} - 3)
\end{aligned}
\tag{2}
$$

2.3 Least squares optimisation of hyperelastic meta-model

$$
\begin{aligned}
\frac{\partial f_{NH}(\lambda)}{\partial \mu} = \frac{\frac{1}{2}(\lambda^2 + 2\lambda^{-1} - 3)}{\Psi_{NH} + 0.001} \\
\frac{\partial f_{MR}(\lambda)}{\partial C_1} = \frac{\lambda^2 + 2\lambda^{-1} - 3}{\Psi_{MR} + 0.001} \\
\frac{\partial f_{MR}(\lambda)}{\partial C_2} = \frac{2\lambda + \lambda^{-2} - 3}{\Psi_{MR} + 0.001} \\
\frac{\partial f_{O_1}(\lambda)}{\partial \mu_i} = \frac{\frac{1}{\alpha_i}(\lambda^{\alpha_i} + 2\lambda^{-\frac{\alpha_i}{2}} - 3)}{\Psi_{O_1} + 0.001} \\
\frac{\partial f_{O_1}(\lambda)}{\partial \alpha_i} = \frac{\frac{\mu_i}{\alpha_i}(\lambda^{\alpha_i} * ln\lambda - \lambda^{-\frac{\alpha_i}{2}} ln\lambda) - \frac{\mu_i}{\alpha_i^2}(\lambda^{\alpha_i} + 2\lambda^{-\frac{\alpha_i}{2}} - 3)}{\Psi_{O_1} + 0.001}
\end{aligned}
\tag{3}
$$

2.4 Stability conditions

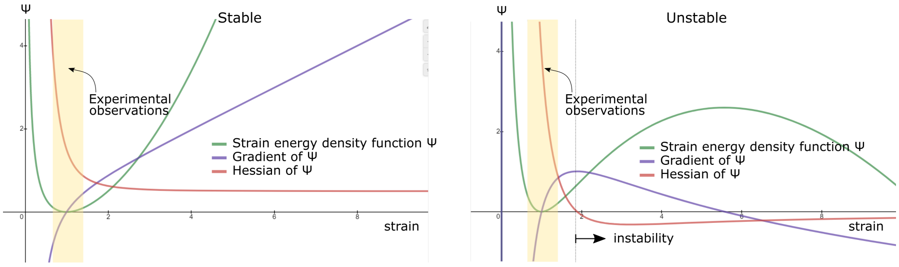

Fig. 2 Stability conditions of hyperelastic functions. A stable (left) and an unstable (right) function. Shaded area indicates region where parameters are reported in the literature. However, simulation may exhibit deformation over wider strains (see Fig. 7 in [5]).

Table 2 Stability conditions of hyperelastic strain energy density functions (Ψ) at rest ($\lambda_i = 1$), and during compression ($\lambda_i < 1$) and tension ($\lambda_i > 1$).

**Function**	**State**	**Condition**	**Range**
$\Psi(\lambda_1, \lambda_2, \lambda_3)$	Resting	$\Psi = 0$<br>$\frac{\partial \Psi}{\partial \lambda_i} = 0$<br>$det[\frac{\partial^2 \Psi}{\partial \lambda_i \partial \lambda_j}] > 0$	$\lambda_1 = 1$<br>$\lambda_2 = 1$<br>$\lambda_3 = 1$
	Compression	$\lim_{\lambda_i \to 0} \Psi = \infty$<br>$\lim_{\lambda_i \to 0} \frac{\partial \Psi}{\partial \lambda_i} = -\infty$<br>$\frac{\partial \Psi}{\partial \lambda_i} < 0$	$0 < \lambda_i < 1$
	Tension	$\lim_{\lambda_i \to \infty} \Psi = \infty$<br>$\lim_{\lambda_i \to \infty} \frac{\partial \Psi}{\partial \lambda_i} = \infty$<br>$\frac{\partial \Psi}{\partial \lambda_i} > 0$	$1 < \lambda_i < \infty$
	All	$\frac{\partial^2 \Psi}{\partial \lambda_i^2} > 0$	$0 < \lambda_i < \infty$

2.5 Optimisation of meta-models

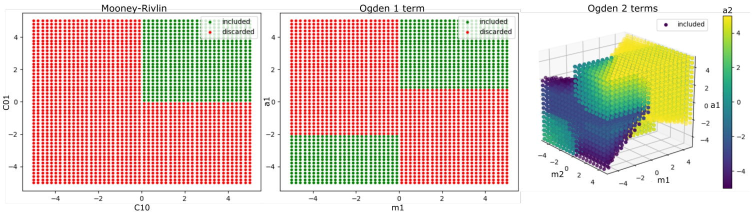

Fig. 3 Meta-model parameters search space.

Table 3 Elapsed time of key simulation steps of the deformation of a synthetic phantom. Average time and standard deviation (within parenthesis) are shown in milliseconds for single tissue (st) and multiple tissue (mt) experiments of two volumetric mesh resolution (see Fig. 3 (left) in main manuscript). Simulation was performed on an Alienware 13 R3 laptop with Intel Core i7 CPU @ 2.8GHz, 32GB RAM, x64 Windows 10 OS, and NVIDIA GeForce GTX 1060 with 6GB GPU.

			Main loop (in ms)				
		**Pre-processing**	**Compute internal forces**	**Assemble system**	**Solve system**	**Time Integration**	**Main loop**
**st**	**coarse**	569	27.44 (3.9)	18.32 (2.0)	63.36 (4.9)	4.95 (5.4)	118.78 (10.2)
**st**	**fine**	1026	36.40 (3.6)	28.31 (2.5)	90.4 (5.3)	4.16 (1.2)	164.20 (8.9)
**mt**	**coarse**	2577	70.93 (5.2)	55.43 (3.6)	174.03 (6.1)	5.91 (7.6)	310.90 (11.8)
**mt**	**fine**	9953	126.99 (14.9)	136.8 (5.6)	437.54 (9.5)	5.27 (1.4)	710.87 (17.0)

3 Gaussian Process

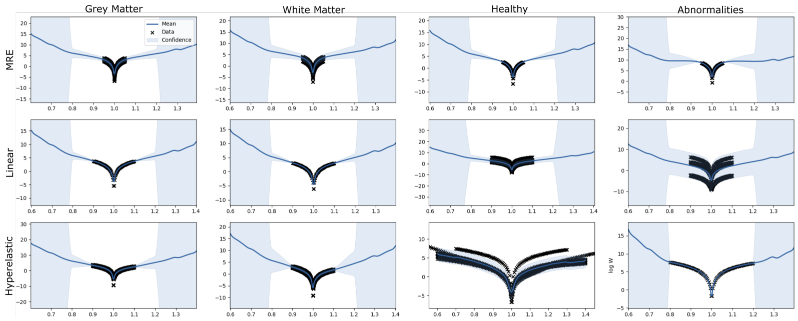

Fig. 4 Gaussian Process (log space) of strain energy density functions extrapolated over wider ranges of stretches.

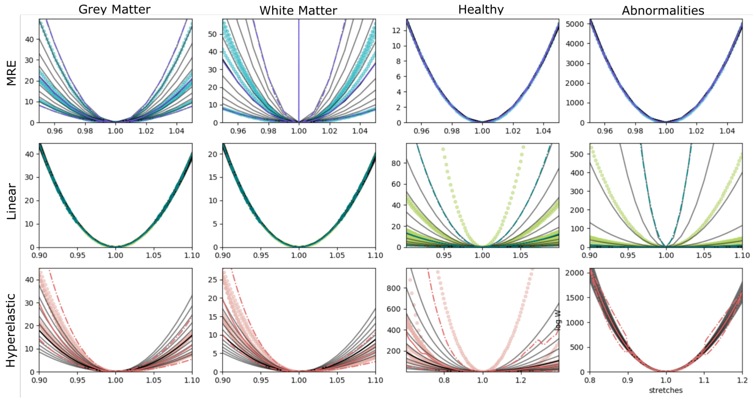

Fig. 5 GP distributions and sampling of strain energy density functions.

4 Simulation

Table 4 Physical properties of cases and parameters used for simulation. Young's modulus $E = 2\mu(1 + v)$ and Lamé coefficient $\lambda = \frac{Ev}{(1+v)(1-2v)}$ are using Poisson's ratio $v = 0.45$. Total mass is computed based on density $\rho = 1211.5$. Implicit backward Euler integration is executed with time step $h = 0.001$. Rayleigh matrix $\mathbf{D} = k_d\mathbf{K} + m_d\mathbf{M}$, where $k_d = 0.001$ and $m_d = 0.0$ are stiffness and damping coefficients, respectively.

	**Physical properties**				
**case**	volume $[10^{-3}m^3]$	mass $[Kg]$	density $[Kg/m^3]$	shear modulus $[Pa]$	Poisson's ratio v
Synthetic	1.6092	1.9495	1211.5	Sampled from generative model	0.45
0529	1.1654	1.4119			
0614	1.0617	1.2862			
0660	1.1344	1.3743			
0685	1.0899	1.3204			
0535	1.2388	1.5008			
0555	1.1706	1.4182			
0603	1.0601	1.2843			
0684	1.1865	1.4374			

Table 5 Geometrical properties of clinical cases used for simulation. For each case, we report the total number of nodes and elements, as well as the number of nodes along ventricles and resection margins that are used to compute RMSE (our similarity metric). Median and median absolute deviation are shown in the last row.

**patient**		**volume**			
		elements		similarity nodes	
case	temporal lobe	single tissue	multi tissue	ventricles	resection
0529	left	3619	5060	263	26
0614	left	2692	4112	196	26
0660	left	2795	4308	198	32
0685	left	2425	4093	164	30
0535	right	2662	4225	174	27
0555	right	2221	3932	140	30
0603	right	2742	4348	169	42
0684	right	2250	3930	137	38
	**median (MAD)**	2677 (185)	4168.5 (159.5)	171.5 (25.5)	30 (3.5)

5 Synthetic brain phantom

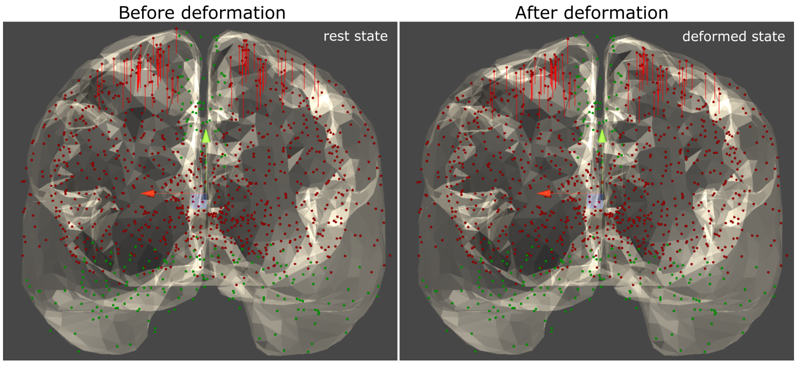

Fig. 6 Validation on MNI phantom. Nodes under deformation (red circles) and boundary conditions (green circles) are of brain tissue (translucent). An external force of 10 N (red vertical lines) is applied for compression to a subset of nodes located superiorly.

6 Temporal lobe resection cases

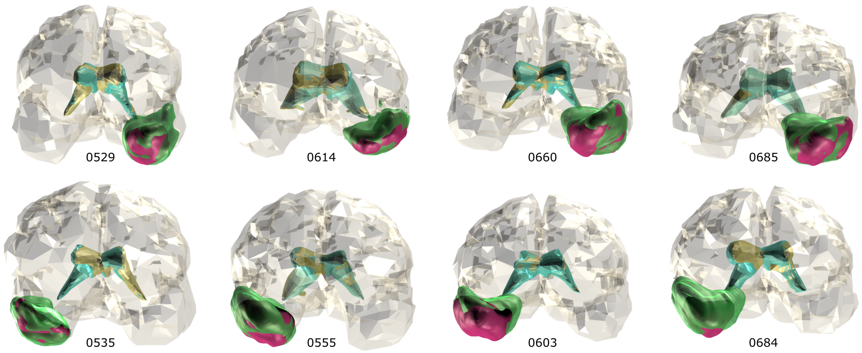

Fig. 7 Application to account for brain shift in temporal lobe resection cases. *Rest state* of ventricles (in yellow) and manually-delineated resection mask (in green) in pre-operative images. *Deformed stated* of ventricles (in blue) and automatedly-segmented resection cavity (in purple) from post-operative images.

Acknowledgements This research was funded/supported by the Health Innovation Challenge Fund (WT106882), the Wellcome/EPSRC Centre for Medical Engineering [WT203148/Z/16/Z], and the National Institute for Health Research (NIHR) Biomedical Research Centre based at Guy's and St Thomas' NHS Foundation Trust and King's College London and/or the NIHR Clinical Research Facility. We are grateful to the Wolfson Foundation and the Epilepsy Society for supporting the Epilepsy Society MRI scanner. The views expressed in this publication are those of the authors and not necessarily those of the Wellcome Trust, NHS, the NIHR or the Department of Health. **Ethical Approval** All data were evaluated retrospectively.

All studies involving human participants were in accordance with the ethical standards of the institutional and/or national research committee and with the 1964 Helsinki declaration and its later amendments or comparable ethical standards. **Informed Consent** For this type of study formal consent is not required.

Conflict of interest. The authors declare that they have no conflict of interest.

References

1. Budday, S., Sommer, G., Birkl, C., Langkammer, C., Haybaeck, J., Kohnert, J., Bauer, M., Paulsen, F., Steinmann, P., E.Kuhl, Holzapfel, G.A.: Mechanical characterization of human brain tissue. Acta Biomaterialia **48**, 319–340 (2017)
2. Mihai, L.A., Budday, S., Holzapfel, G.A., Kuhl, E., Goriely, A.: A family of hyperelastic models for human brain tissue. J Mech and Physics Solids **106**, 60–79 (2017)
3. Mihai, L.A., Chin, L., Janmey, P.A., Goriely, A.: A comparison of hyperelastic constitutive models applicable to brain and fat tissues. Royal Soc **12**, 1–12 (2015)
4. Morin, F., Chabanas, M., Courtecuisse, H., Payan, Y.: Biomechanical modelling of brain soft tissues for medical applications. Biomech Living Organs pp. 127–146 (2017)
5. Xu, H., Sin, F., Zhu, Y., Barbic, J.: Nonlinear Material Design Using Principal Stretches. ACM Transactions on Graphics pp. 1–11 (2015)
